# Six Clinical Cases

**Published:** 1891-06

**Authors:** F. H. Lutze

**Affiliations:** Cheshire, New York


### SIX CLINICAL CASES.

F. H. LUTZE, M. D., CHESHIRE, NEW YORK.

I. HEADACHE.—Miss H., æt. seventeen years, robust, sanguine temperament, suffered from severe headache, worse in the morning, face red and bloated; no other symptoms. Had been treated in Illinois by an old-school doctor without any benefit.

Nux-vom.$^{2c}$, a powder every third day till three were taken, and S. L. As she did not return, I called at her residence three weeks later, and heard that the headaches had been entirely relieved at first, but later returned worse than ever; thought there was no use taking any more medicine.

From her aunt I received the following additional symptoms: Miss H. had been suffering in the West from chorea, but had been cured (?) by the old-school doctor there; then the headache came on, which he could not cure. She was afraid in the dark, when going to bed had to look with the light under the bed, in the closets and dark corners, before she could go to sleep. If free from headache, it would begin at any time on being startled or frightened. On attempting to set a plate on a high shelf yesterday the plate slipped, and though she caught it again without damage, a most terrible headache was the result. She cannot think quick enough when speaking, which causes stammering. Menses regular, no pain, but flow rather watery. Stram.$^{1m}$ and S. L. She never had another headache, no fear in the dark, no stammering; menses normal, in short, feels better in every respect than ever before.

There is no doubt that the whole diseased condition resulted from the suppressed chorea, Nux-v. antidoting the effects to some extent, at least; the aggravation followed, yet it required but one dose of the proper remedy in high potency to cure.

II. BACKACHE.—Miss T., a school-teacher, has been suffering for years from a pain in the sacrum, with constant nausea. She is never free from it; feels it at night on awaking from sleep. She has been under old school-treatment now and then, but without any benefit; she can give no other symptoms, and looks apparently healthy. She received S. L. with instructions how to observe symptoms, and to call again in a week. Then she reported the following additional symptoms:

Backache worse riding in a carriage; always with nausea. Fullness to bursting on the left side of the spine; all these symptoms are worse since taking the medicine—Sac-Lac! (This is no doubt due to trying to observe new symptoms, whereby the attention was drawn more to the suffering.) Does not urinate from morning till night on going to bed, and even then the urine is scanty. On repeated trials the quantity for the twenty-four hours less than a pint of urine; color and specific gravity normal; reaction nearly neutral, tests showed only some phosphates. Every morning on arising the tongue has a thick yellow coat on the base. Kali-bichrom.$^{1m}$, one powder, and S. L.

After a week she wrote: "I feel a great deal better in every respect, please send some more of the same medicine." I sent S. L.

Three weeks later she reported not quite so well. Headache in forehead and temples, affecting the vision, sometimes awakens with it in the morning; again does not come till the afternoon. Flushes of heat; hands and feet cold. Kali-bichrom$^{75m}$, one powder, and S. L.

Three months later I met her; she looked very much improved, and said she was perfectly well.

Colocynthis, Psorinum, Pulsatilla, Physostigma, and Zingiber are the only remedies I could find, having the peculiar symptom: "Backache with nausea." Had I given either of these remedies this symptom might have been cured, and perhaps not, but certainly not the patient, and the case would no doubt have been complicated thereby. The symptom worse by riding in a carriage is sometimes cured by a remedy having worse from motion or jarring. I have seen it disappear under Bell. Yet Bœnninghausen gives Kali-carb. among the remedies covering the symptom, and Kali-bichrom. has no doubt the same.

III. HEADACHE.—Miss Hattie P. came about a year ago to be cured of her headache. She said it was a constant companion, she had it day and night; occupied the whole head, was more severe now and then, but she could give no time or conditions of aggravation or amelioration. Menses regular, but always accompanied with pains in the uterine region for the first two or three days, which were often so severe that she had to go to bed. Between the periods a constant heavy pressing-down pain in the sacrum.

Objective symptoms: The red color of the hands and face had a decided bluish tinge, and the skin was very rough. The whites of the eyes appeared as if painted with yellow ochre.

She had been under old-school treatment some, but with no benefit; had been examined by an oculist, who said her trouble was due to astigmatism, and prescribed spectacles; these ameliorated the headache for a time, but had no influence upon backache or menstrual pains. Bell.$^{cm}$, one powder, and another powder one month later, which cured all the disease symptoms, so that she even found no more use for her spectacles.

Lippe's *Repertory* mentions about thirty-five remedies having yellowness of the conjunctiva; but the other symptoms pointed unerringly to Bell. as the simillimum, and the result proved the correctness of the choice, and it required but two doses to cure the entire diseased condition, which had existed for many years, and which allopathic drugging had not benefited in the least. I am also convinced that even Bell. in a lower potency, and often repeated, would only have produced an aggravation, not a cure.

IV. WORM FEVER.—Arthur H., æt. three years, has been a pale, feeble child since birth; never had a normal stool, but always diarrhœa, generally with prolapse of rectum. Awakened, or, at least, sits up at night in bed screaming, and cannot be pacified; wets the bed at night; also passes worms now and then. I had treated the child now and then, giving Cina$^{200}$, which improved him very much, but finally the mother brought him to me, saying he had the worst worm fever he had ever had, though he had this every now and then. She could give no new symptoms. The boy's cheeks and tips of ears were a brilliant scarlet red, the other parts of the face, especially around the mouth, white as snow; brilliant staring eyes, dilated pupils. Skin dry and hot like fire. When I spoke to him coaxingly he flew in a rage, such as I should have thought a child so young hardly capable of.

Bell.$^{cm}$, one powder in water, a teaspoonful every hour, produced such a remarkable improvement in one day that he seemed almost well, but on the third day there was some return of the fever and irritability, when I gave a small dose of Bell.$^{m}$ (Fincke), which cured in a week the whole condition, and he has been well and healthy ever since.

The appearance of the face, as described above, I have often noticed in children, and found this objective symptom always a good indication for Bell., so that I never hesitate to give Bell. in the highest potencies, and have always found it to act promptly.

Apis has a somewhat similar objective symptom, which I have verified several times, but here the color is not scarlet, but a bright pinkish hue, often extending from one cheek downward across the chin, more or less, and again upward on the other cheek. The ears are white like the rest of the face, but do not appear as white as under Bell., on account of the paler pinkish-red. I consider such objective symptoms very valuable, especially in practice among the less intelligent classes of people, who are less observant, and often even become irritated when asked many questions. I gave Apis in two cases of hydrocephalus mainly on these indications, with excellent results.

V. Sciatica.—Mr. H., æt. forty-two, has been all of nine months under treatment, first allopathic, then with two eclectic physicians successively, for a pain in the right leg and hip, which I called sciatica. When he came to see me he presented the following symptoms :

Pain in right hip and lower extremity, better from continued exercise, especially running, till tired, then better for awhile by lying still; better from heat and rubbing; worse in damp weather *and in the daytime;* at night in bed he can turn over and roll around freely without suffering any pain. Rhus$^{75m}$, one powder, although it does not correspond to the aggravation in the daytime, yet every other symptom seemed to correspond.

The next day I was sent for, as he was very much worse, yet the night had been passed as comfortable as usual. He showed me some homœopathic pellets the last eclectic doctor had given him, a one-half ounce vial nearly full, with about a half drachm of a tincture looking very much like Rhus-tox., and concluded that the symptoms presented the day before were all due to this drug, for the Rhus$^{75m}$ had removed them all, except the amelioration at night, which continued along with the following new symptoms : Pain in the right hip and lower extremity of a drawing, tearing, cramping character, with a sensation of contraction or shortening ; drawing aching on inner side of the left thigh, all worse in the daytime and from slightest motion; better from lying perfectly still and at night; can move and roll around in bed at night freely, without any pain. Bowels constipated, has to urge a great deal to expel the dry, hard stool, this as also coughing, sneezing, or stooping forward, aggravates the pain. Colocynth.$^{200}$, one powder and S. L.

After a week there was only a slight improvement. Colocynth.$^{200}$, a powder daily for a week. Now the improvement was marked. Coloc.$^{200}$, a powder daily for two weeks. Very much improved again. S. L. for two weeks. Reports about well, but the stool is still dry, requires much urging, and causes same pain at anus on voiding. There is an oozing from the anus, keeping the perinæum and coccyx moist. Anus is surrounded with a pimply eruption. Sepia$^{cm}$ (F.) completed the cure. I am inclined to think the daily repetition of the dose would not have been necessary if I had had a higher potency of Colocynth to give. The following case likewise shows the better action of the higher potency:

VI. Facial Neuralgia.—Mrs. L. Neuralgia on left side of face, neck, and left shoulder; better from warmth of fire, rubbing, and external hot applications and motion, must move or rock, cannot keep still; worse in the morning at nine A. M., and evening from eight to twelve, from rest or cold. Picking or pressing with a toothpick at and between the teeth on the left side also relieves somewhat. Sleepy after the aggravation. Rhus$^{200}$, and next day Rhus$^{1m}$, no change. S. L. for three days, but getting worse. On the sixth day I gave in the morning Rhus$^{105m}$, one dose in water, a spoonful every hour, and a cure followed in four hours. The neuralgia returning a few days later, another small dose of the same, Rhus$^{105m}$, was given, which put an end to the trouble.

